# A Mitotic Phosphorylation Feedback Network Connects Cdk1, Plk1, 53BP1, and Chk2 to Inactivate the G_2_/M DNA Damage Checkpoint

**DOI:** 10.1371/journal.pbio.1000287

**Published:** 2010-01-26

**Authors:** Marcel A. T. M. van Vugt, Alexandra K. Gardino, Rune Linding, Gerard J. Ostheimer, H. Christian Reinhardt, Shao-En Ong, Chris S. Tan, Hua Miao, Susan M. Keezer, Jeijin Li, Tony Pawson, Timothy A. Lewis, Steven A. Carr, Stephen J. Smerdon, Thijn R. Brummelkamp, Michael B. Yaffe

**Affiliations:** 1 David H. Koch Institute for Integrative Cancer Research, Massachusetts Institute of Technology, Cambridge, Massachusetts, United States of America; 2 Cellular and Molecular Logic Team Integrative Network Biology initiative (INBi) Section of Cell and Molecular Biology, The Institute of Cancer Research, London, United Kingdom; 3 Departments of Biological Engineering and Biology, Massachusetts Institute of Technology, Cambridge, Massachusetts, United States of America; 4 Broad Institute of Harvard and MIT, Cambridge, Massachusetts, United States of America; 5 Samuel Lunenfeld Research Institute, Mount Sinai Hospital, Toronto, Canada; 6 Cell Signaling Technologies, Danvers, Massachusetts, United States of America; 7 Division of Molecular Structure, Medical Research Council (MRC) National Institute for Medical Research, London, United Kingdom; 8 Whitehead Institute, Massachusetts Institute of Technology, Cambridge, Massachusetts, United States of America; National Cancer Institute, United States of America

## Abstract

DNA damage checkpoints arrest cell cycle progression to facilitate DNA repair. The ability to survive genotoxic insults depends not only on the initiation of cell cycle checkpoints but also on checkpoint maintenance. While activation of DNA damage checkpoints has been studied extensively, molecular mechanisms involved in sustaining and ultimately inactivating cell cycle checkpoints are largely unknown. Here, we explored feedback mechanisms that control the maintenance and termination of checkpoint function by computationally identifying an evolutionary conserved mitotic phosphorylation network within the DNA damage response. We demonstrate that the non-enzymatic checkpoint adaptor protein 53BP1 is an in vivo target of the cell cycle kinases Cyclin-dependent kinase-1 and Polo-like kinase-1 (Plk1). We show that Plk1 binds 53BP1 during mitosis and that this interaction is required for proper inactivation of the DNA damage checkpoint. 53BP1 mutants that are unable to bind Plk1 fail to restart the cell cycle after ionizing radiation-mediated cell cycle arrest. Importantly, we show that Plk1 also phosphorylates the 53BP1-binding checkpoint kinase Chk2 to inactivate its FHA domain and inhibit its kinase activity in mammalian cells. Thus, a mitotic kinase-mediated negative feedback loop regulates the ATM-Chk2 branch of the DNA damage signaling network by phosphorylating conserved sites in 53BP1 and Chk2 to inactivate checkpoint signaling and control checkpoint duration.

## Abstract 2

A combined computational and biochemical approach reveals how mitotic kinases allow cell division to proceed in the presence of DNA damage.

## Author Summary

DNA is constantly damaged both by factors outside our bodies (such as ultraviolet rays from sunlight) and by factors from within (such as reactive oxygen species produced during metabolism). DNA damage can lead to malfunctioning of genes, and persistent DNA damage can result in developmental disorders or the development of cancer. To ensure proper DNA repair, cells are equipped with an evolutionarily conserved DNA damage checkpoint, which stops proliferation and activates DNA repair mechanisms. Intriguingly, this DNA damage checkpoint responds to DNA damage throughout the cell cycle, except during mitosis. In this work, we have addressed how cells dismantle their DNA damage checkpoint during mitosis to allow cell division to proceed even if there is damaged DNA present. Using the observation that kinases phosphorylate their substrates on evolutionarily conserved, kinase-specific sequence motifs, we have used a combined computational and experimental approach to predict and verify key proteins involved in mitotic checkpoint inactivation. We show that the checkpoint scaffold protein 53BP1 is phosphorylated by the mitotic kinases Cdk1 and Polo-like kinase-1 (Plk1). Furthermore, we find that Plk1 can inactivate the checkpoint kinase Chk2, which is downstream of 53BP1. Plk1 is shown to be a key mediator of mitotic checkpoint inactivation, as cells that cannot activate Plk1 fail to properly dismantle the DNA damage checkpoint during mitosis and instead show DNA damage-induced Chk2 kinase activation. Two related papers, published in *PLoS Biology* (Vidanes et al., doi:10.1371/journal.pbio.1000286) and *PLoS Genetics* (Donnianni et al., doi:10.1371/journal.pgen.1000763), similarly investigate the phenomenon of DNA damage checkpoint silencing.

## Introduction

Throughout the life of an organism, cellular DNA constantly encounters chemical and radiation-induced damage. Solar and terrestrial sources of radiation, along with the oxidative by-products of normal metabolism, result in chemical modifications of DNA bases and disruption of the sugar phosphate backbone. Additional DNA lesions, including mismatched bases, and single- or double-stranded DNA breaks, also arise during the process of replication, which is not an error-free process [1]. To cope with these types of genotoxic damage, cells activate powerful DNA damage-induced cell cycle checkpoints that coordinate cell cycle arrest with recruitment and activation of the DNA repair machinery [2]–[6]. Depending on the amount of damage and the specific cell type, cross-talk between the checkpoint and repair pathways with pathways involved in programmed cell death leads to the elimination of irreparably damaged cells by apoptosis [7]. The global importance of these cell cycle checkpoint pathways in maintaining genomic integrity is highlighted by the observation that loss, mutation, or epigenetic silencing of checkpoint genes is frequently observed in cancer [1],[4]. Conversely, deletion of checkpoint genes in non-neoplastic cells has been shown to cause genomic instability and predisposition to transformation [1],[4].

Loss of DNA damage checkpoints during early stages of tumorigenesis not only facilitates the acquisition of additional mutations over time [8],[9] but can also be exploited in various forms of human cancer treatment. Radiotherapy as well as many types of anti-tumor chemotherapy are believed to preferentially kill tumor cells by generating extensive amounts of DNA damage that promotes cell death in checkpoint-compromised tumors, but not in the surrounding non-neoplastic tissue where the checkpoint and repair pathways are intact [10]. The primary cytotoxic lesion created by therapeutic radiotherapy and most other genotoxic treatments are DNA double-strand breaks (DSBs). It has been estimated that a single unrepaired DSB is sufficient for cell lethality [11].

Early events following DSB generation include local alterations in chromatin structure, recruitment of the Mre11-Rad50-Nbs1 mediator complex to the DNA, and phosphorylation of the variant Histone H2AX by an initial wave of activation of the checkpoint kinase ATM [2],[12]–[14]. Subsequent recruitment of the protein MDC1 dramatically enhances further local activation of ATM as part of a positive feedback loop, which in turn recruits molecules like 53BP1 and BRCA1 [15]–[17]. 53BP1 facilitates DNA repair by the error-prone non-homologous end joining (NHEJ) pathway [18],[19], while BRCA1 is important for DNA repair by the error-free homologous recombination pathway during the S and G2 phases of the cell [20]. A major target of ATM is the effector kinase Chk2, a critical effector kinase that functions downstream of ATM to arrest the cell cycle after DSBs by inactivating phosphatases of the Cdc25 family through catalytic inactivation, nuclear exclusion, and/or proteasomal degradation [21],[22]. This, in turn, prevents Cdc25 family members from dephosphorylating and activating Cyclin-Cdk complexes, thereby initiating G_1_/S and G_2_/M cell cycle checkpoints.

In order for cells to survive DNA damage, it is important that cell cycle arrest is not only initiated but also maintained for the duration of time necessary for DNA repair. Mechanisms governing checkpoint initiation versus maintenance appear to be molecularly distinct. This was initially demonstrated by the observation that interference with specific checkpoint components can leave checkpoint initiation intact but disrupt checkpoint maintenance, leading to premature cell cycle reentry accompanied by death by mitotic catastrophe [7],[15],[23]–[25]. Although the process of checkpoint termination and cell cycle reentry has not been studied extensively, the existing data suggest that inactivation of a checkpoint response is an active process that requires dedicated signaling pathways, such as the Plk1 pathway [2],[26],[27]. Intriguingly, a number of proteins involved in terminating the maintenance phase of a DNA damage checkpoint also play critical roles during later mitotic events, suggesting the existence of a positive feedback loop in which the earliest events of mitosis involve the active silencing of the DNA damage checkpoint through one or more mechanisms that remain unclear.

Checkpoint silencing has been best studied in the budding yeast *S. cerevisiae* and has revealed several essential genes in this process, for example the phosphatases Ptc2 and Ptc3, Casein kinase-I, and Srs1 [28]–[30]. In addition, the Polo-like kinase Cdc5 is required for silencing checkpoint signaling, and this requirement appears to be widely conserved, since *S. cerevisiae*, *X. Leavis*, and human cells all depend on Plks for silencing of the S-phase or G_2_ checkpoints, respectively [29],[31]–[33]. The activity of Polo-like kinases has been shown to be required for inactivation of the ATR-Chk1 pathway and the Wee1 axis of checkpoint signaling. Specifically, Plk1 was shown to create β-TrCP-binding sites on both Wee1 and the Chk1 adaptor protein Claspin, resulting in efficient ubiquitin-mediated degradation of these target proteins [32]–[36]. Thus far, only inactivation of checkpoint components of the ATR-Chk1-Wee1 signaling axes has been identified in relation to maintenance and termination of cell cycle checkpoints. DSBs, however, primarily trigger a checkpoint arrest through the ATM-Chk2 signaling pathway. How, and if, the ATM-Chk2 signaling axis is actively silenced during release of the G2 DNA damage checkpoint is currently unclear. Here, we analyzed potential feedback mechanisms responsible for terminating this process. We reasoned that inactivation of cell cycle checkpoints after DSBs should involve at least two arms of the ATM-Chk2 checkpoint response—both the upstream sensor arm that maintains activation of ATM and the downstream effector arm that functions at and below the level of Chk2 must be silenced in order to facilitate cell cycle reentry. By using a combination of evolutionarily constrained bioinformatics analysis together with cell cycle–specific modifications of the highly conserved DNA damage checkpoint signaling network, we identified the Cdk- and Plk1-dependent phosphorylation of 53BP1 and Chk2 as critical checkpoint-inactivating events in the sensor and effector arms of the G_2_/M checkpoint pathway, respectively, that are important for checkpoint termination and cell cycle reentry.

## Results

### The ATM-Chk2 Pathway Is Silenced in Mitosis

**Figure 1:**
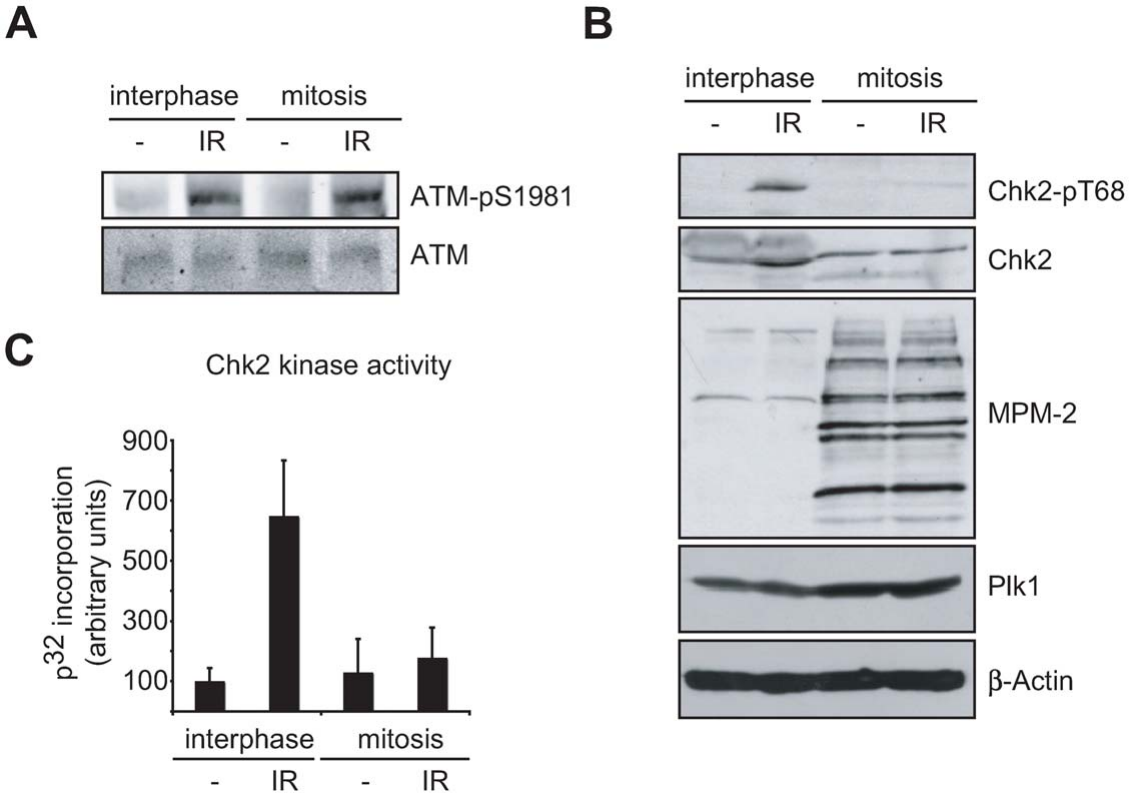
Inactivation of the ATM-Chk2 checkpoint signaling pathway upon mitotic entry. (A) Asynchronous U2OS cells were untreated (“interphase”) or treated with nocodazole (“mitosis”) for 16 h and collected by shake-off. Where indicated, cells were irradiated with 10 Gy and harvested 30 min later. Whole cell lysates were immunoblotted for total and Ser-1981 phosphorylated ATM. (B) Cell lysates prepared as in panel A were immunoblotted with the indicated total and phospho-specific antibodies. (C) Lysates as in panel B were analyzed for Chk2 kinase activity using an IP/kinase assay. Error bars indicate SEM.

To identify potential feedback and control mechanisms that extinguish the ATM-Chk2 signaling axis of the G_2_/M DNA damage checkpoint, we initially investigated whether we could observe silencing of this network under particular cell states or conditions. Molecular targets that are known to be inactivated in other G_2_/M cell cycle checkpoint control pathways, i.e. the ATR/Chk1 pathway, include Wee1 and Claspin [32]–[36] and inactivation of these components results in a shutdown of this checkpoint signaling pathway following mitotic entry. If the ATM-Chk2 pathway was also inactivated upon mitotic entry, clear differences would be expected when interphase cells are compared to mitotic cells following irradiation. To examine this, U2OS cells were exposed to 10 Gy of ionizing radiation (IR), and activation of the upstream checkpoint kinase ATM and the downstream effector kinase Chk2 were examined by immunoblotting (Figure 1A, B). In order to investigate whether mitotic cells remained in mitosis upon irradiation in our experimental set-up, we used two mitotic markers, MPM-2 immunoreactivity and the presence of Plk1 (Figure 1B). The monoclonal MPM-2 antibody was originally cloned on the basis of its ability to specifically recognize mitotic but not interphase cells [37]. MPM-2 recognizes multiple mitosis-specific phospho-proteins, and its reactivity thus indicates the abundance of mitotic cells. Plk1, on the other hand, is highly expressed in G_2_ and M-phase of the cell cycle and is degraded during mitotic exit [38]. Importantly, we observed that irradiation of mitotic cells did not lead to mitotic exit, as judged by the persistently elevated levels of Plk1 and MPM-2 immunoreactivity (Figure 1B). As shown in Figure 1A, in response to IR, ATM was efficiently activated regardless of cell cycle phase. We observed both rapid phosphorylation of Chk2 on Thr-68, a known ATM phosphorylation site, and enhanced Chk2 kinase activity (Figure 1B,C), after irradiation of interphase cells. However, irradiation of mitotic cells failed to result in phosphorylation of Chk2 on Thr-68, and the DNA damage-induced increase in Chk2 kinase activity was severely impaired (Figure 1B,C). This suggests that ATM may not efficiently form complexes with some of its critical downstream substrates such as Chk2 in response to DNA damage during mitosis, resulting in a failure to activate Chk2 and Chk2-dependent effector pathways required for cell cycle arrest. This hypothesis is in line with previous reports in which γ-irradiation or treatment with topoisomerase inhibitors were shown not to interfere with progression of cells already in mitosis [39],[40], indicating that DNA damage checkpoint pathways are functionally inactivated during mitosis.

### Reconstructing a Phosphorylation Network of DNA Damage Proteins

**Figure 2:**
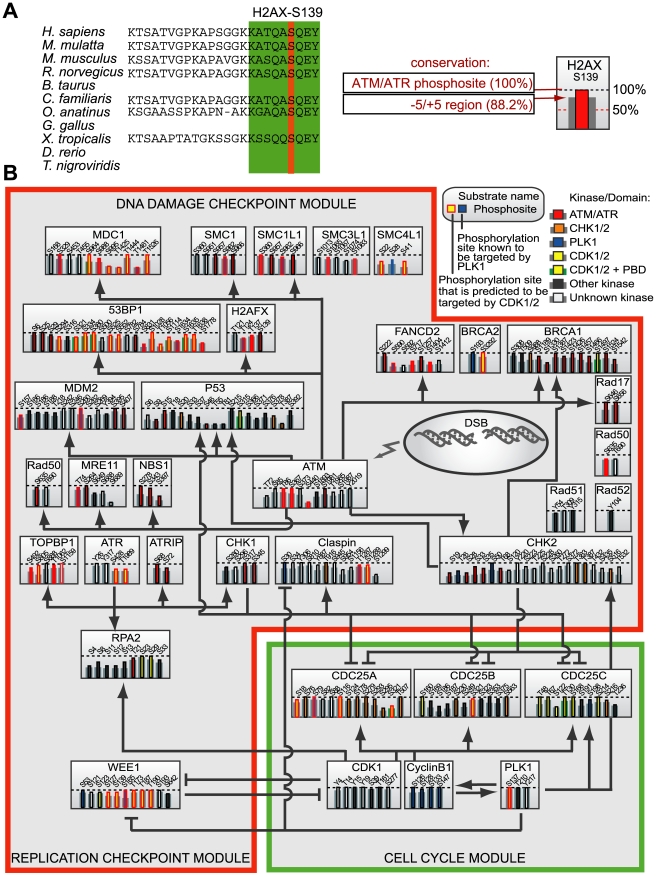
Conservation of mapped phosphorylation sites in the DNA damage signaling network. (A) Example of a conserved ATM/ATR phosphorylation motif [ST]Q in H2AX. Left: the human H2AX sequence, in which the mapped phosphorylation site was identified, was aligned with orthologous sequences from the indicated genomes. No orthologues for cow, chicken, zebrafish, or pufferfish are present in the Ensembl database. Analysis of the −5/+5 region surrounding Ser139 (green box) showed conservation of 100% (*M. mulatta*; *C. familiaris*), 87.5% (*M. musculus*; *R. norveticus*), 75% (*O. anatinus*), and 62.5% (*X. tropicalis*), leading to a mean conservation of 88.2%. Right: the site is indicated by a vertical column composed of central and flanking bars. The height of the central bar indicates the extent of conservation of the central phospho-acceptor residue among the identified orthologues (red, 100%). The height of the flanking bars indicates conservation within the 11 amino acid region surrounding the phosphosite (grey, 88.2%). (B) Phosphorylation network and evolutionary analysis for components of the DNA damage checkpoint signaling pathways. Each protein in the reconstructed network is shown as a grey box containing columns corresponding to each previously identified in vivo phosphorylation sites. The height of the bars in each column indicates the evolutionary conservation of the site amongst the vertebrates, as shown in panel A and tabulated in Table S1. The NetworKIN algorithm was used to reconstruct a network of kinases involved in these phosphorylation events, and predictions for Cyclin-dependent kinases (yellow), ATM/ATR (red), CHK1/2 (orange), and Polo-like kinase-1 (blue) are displayed. Sites known to be phosphorylated by other or unknown kinases are shown in dark and light grey, respectively. Polo-box binding sites are shown in green. Lines indicate established signaling interactions.

To elucidate potential molecular mechanisms responsible for checkpoint silencing of the ATM-Chk2 axis in mitosis, we used a supervised computational network/bioinformatics approach. First, we identified a set of core proteins involved in the human G_2_/M checkpoint and mapped known in vivo phosphorylation sites [41]–[44] onto them (Figure 2A,B and Table S1). Next, this set of phospho-proteins was used to query for conservation of the phosphorylation sites, defined by five residues N-terminal and five residues C-terminal that flank the mapped phospho-residue, in protein orthologs across eleven vertebrate genomes. We computed the conservation as the mean percentage of conserved residues within this eleven-mer site window across these vertebrate genomes. The kinases responsible for generating these phosphorylation sites were identified using data from PhosphoELM [42] or predicted using the NetworKIN algorithm [45]–[47]. In addition, we used Scansite [48] to identify potential docking sites for the Plk1 Polo-Box Domain (PBD) [44],[49],[50] within the network. As would be expected, we observed that many of the checkpoint proteins contained highly conserved ATM/ATR sites (Figure 2A,B and Table S1). Importantly, we also identified highly conserved phosphorylation sites for Cdk1/2 and Plk1 kinases distributed relatively equally on proteins throughout the network, independently of whether the proteins were classified into “checkpoint” or “cell cycle” modules. No potential molecular targets could be uniquely pinpointed by looking only at the putative kinase-substrate level; thus the mitotic/DNA damage phosphorylation network seems to be robust in the sense that they are highly connected via relatively few but pleotropic kinases. However, when we searched for PBD binding sites, only a few network components appeared (Figure 2B) including the previously validated Plk1 binding target Cyclin B [51]. In addition, several components of the checkpoint signaling pathway appeared as putative Plk1 PBD-binding targets, notably MDC1 and 53BP1. Surprisingly, these two proteins belong to the non-enzymatic checkpoint adaptor family of proteins that function in the ATM-Chk2 pathway [16],[52]–[57].

### 53BP1 Is a Target for Cdk1 and Plk1 and Fails to Form Foci after DNA Damage in Mitosis

**Figure 3:**
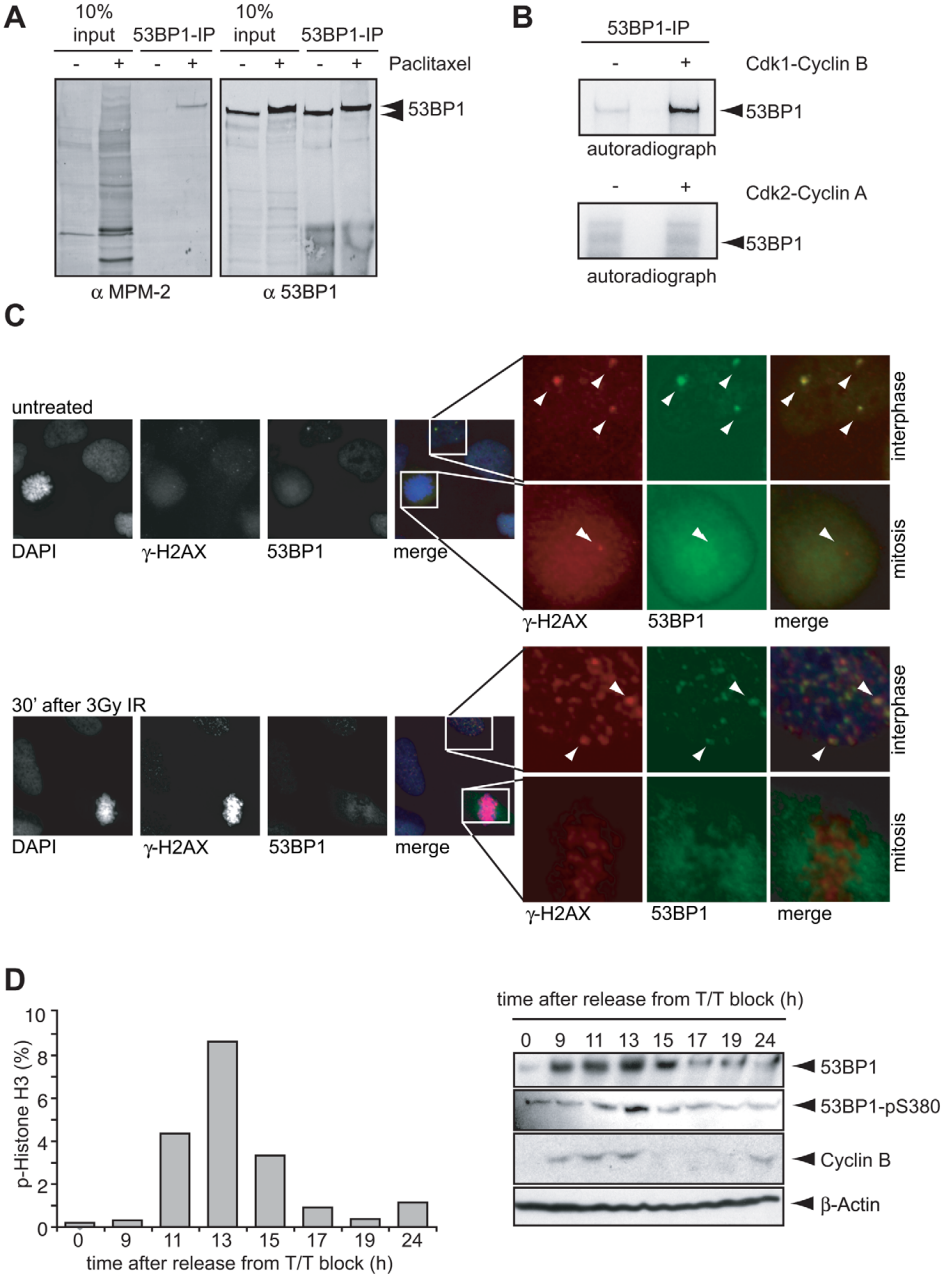
Cell cycle–dependent changes in post-translational modification and localization of 53BP1. (A) Asynchronous U2OS cells were left untreated or treated with paclitaxel for 16 h and collected by shake-off. Cell lysates were used for 53BP1 immunoprecipitations and analyzed by Western blotting. Ten percent of input as well as 53BP1 immunoprecipitations were analyzed using anti-MPM-2 and anti-53BP1 antibodies. (B) Interphase U2OS cells were lysed and processed for 53BP1 immunoprecipitations. 53BP1 immunoprecipitations were subsequently used as in vitro substrates for either Cdk1-Cyclin B or Cdk2-Cyclin A. Phosphorylation of 53BP1 was analyzed by SDS-PAGE/autoradiography. (C) Asynchronously growing U2OS cells were left untreated or irradiated with 5 Gy ionizing radiation. Thirty minutes after irradiation, cells were fixed, permeabilized, and stained for 53BP1 and γ-H2AX. Magnifications represent examples of mitotic or interphase cells before or after irradiation. (D) U2OS were synchronized using a double thymidine block. At indicated time points after release, cells or cell lysates were analyzed by flow cytometry or SDS-PAGE/immunoblotting as indicated. Left panel: at each time point, 10,000 events were analyzed for phospho-Histone H3 staining by flow cytometry. Right panel: lysates prepared at indicated time points were analyzed for expression of 53BP1, phosphoSer380-53BP1, β-actin, and Cyclin B.

We focused on 53BP1, since our analysis predicted eight highly conserved Cdk1/2 phosphorylation sites as well as three sites with lower conservation. Importantly, five of the highly conserved Cdk1/2 phosphorylation sites constitute putative PBD binding sites. We have previously shown that 53BP1 is a target of Cdk1-Cyclin B during mitosis [45]. Here, we aimed to investigate the functional implications of these phosphorylation events and again employed the MPM-2 antibody, which recognizes proteins that are phosphorylated on Cdk1/2 consensus motifs [37],[58],[59]. By immunoprecipitating 53BP1 from mitotic cell extracts, we observed clear immunoreactivity with the MPM-2 antibody, in stark contrast to 53BP1 immunoprecipitated from interphase cells (Figure 3A). These results were further strengthened by in vitro kinase assays, in which recombinant Cdk1-Cyclin B, but not Cdk2-CyclinA, efficiently phosphorylated 53BP1 (Figure 3B).

If 53BP1 is a critical target for checkpoint silencing by mitotic kinases, then the function of 53BP1 should be altered during mitosis. We therefore investigated the co-localization of 53BP1 and DNA damage–induced foci at different cell cycle phases. Few γ-H2AX foci were observed in untreated cells, while their number increased dramatically after 3Gy of IR (Figure 3C, Figure S1B left panel). Similar behavior was observed for 53BP1 (Figure 3C, Figure S1B left panel). In interphase cells, approximately 70% of γ-H2AX foci contained 53BP1 (Figure 3C, Figure S1B middle panel). However, when nuclear foci of 53BP1 were present, they always overlapped with γ-H2AX in both untreated as well as in IR-treated cells during interphase (Figure 3C, Figure S1B right panel). In contrast, in mitotic cells there were essentially no distinct 53BP1 foci that were observed regardless of the presence or absence of irradiation, and instead 53BP1 appeared to be largely excluded from chromatin. Hence, in mitosis no overlap was detected between the localization of γ-H2AX foci and 53BP1, showing that the function of 53BP1 in the DNA damage response (DDR) is indeed modified during mitosis, either directly or indirectly (Figure 3C).

In addition to changes in IR-induced localization of 53BP1, we also observed that the protein levels of 53BP1 rapidly declined as cells passed synchronously through the cell cycle (Figure 3D). However, the decrease in 53BP1 protein levels occurred only at late stages of mitosis or cell cycle reentry, after the destruction of Cyclin B, and thus may not have a prominent role in G_2_ checkpoint inactivation.

**Figure 4:**
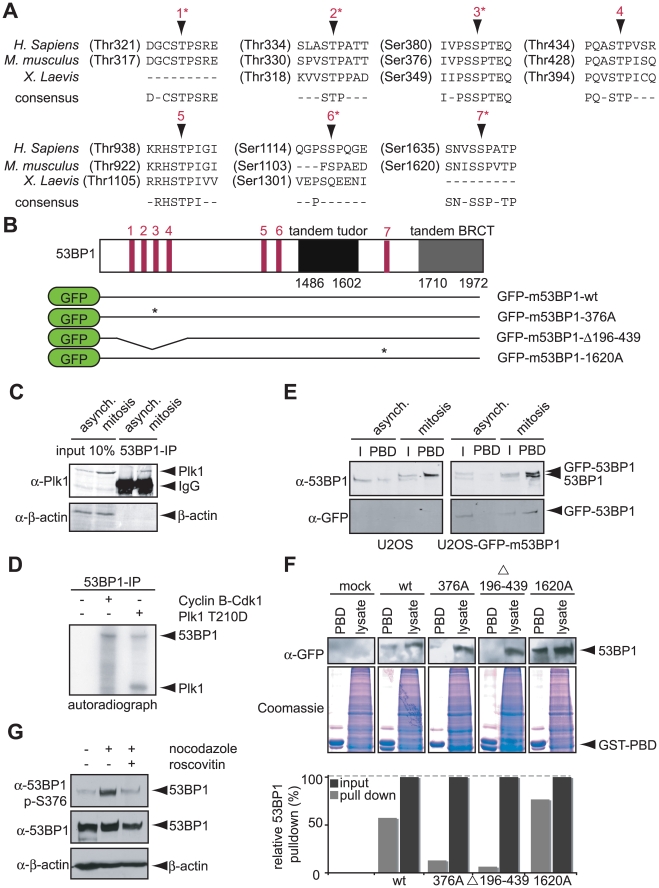
Interaction of 53BP1 and Plk1. (A) Putative Polo-like kinase-1 binding sites within 53BP1 are indicated, along with site conservation across *M. musculus* and *X. tropicalis*. Asterisks mark residues that were found to be phosphorylated in vivo. (B) Schematic representation of 53BP1 protein organization along with location of putative Plk1-binding sites. Lower part: a selection of GFP-tagged murine 53BP1 constructs used in this study. Asterisks mark residues that were mutated to Ala. (C) U2OS cells were left untreated or treated with paclitaxel for 16 h, and mitotic cells were isolated by mitotic shake-off. 53BP1 was immunoprecipitated, and lysates (“input 10%”) or immunoprecipitations (“53BP1 IP”) were analyzed by Western blotting for 53BP1, Plk1, and β-actin. (D) 53BP1 was immunoprecipitated from interphase lysates and used as a substrate for Cdk1-Cyclin B or Plk1 kinase (Plk1 T210D). Incorporation of [^32^P]-γ-ATP was visualized by SDS-PAGE/autoradiography. (E) Interphase or mitotic lysates of U2OS cells and U2OS cells, stably expressing GFP-tagged wt-m53BP1, were incubated with immobilized GST-Plk1-PBD. Endogenous 53BP1 and GFP-tagged m53BP1 associated with GST-Plk1-PBD were analyzed by immunoblotting using anti-GFP and anti-53BP1 antibodies. “I” indicates 10% input for immunoprecipitations. “PBD” indicates pull-downs using the GST-Plk1 Polo-box domain. (F) Mitotic lysates of U2OS cell lines, stably expressing the indicated GFP-tagged m53BP1 constructs, were incubated with immobilized GST-Plk1-PBD. The inputs (“lysate”) and GST-Plk1-PBD associated 53BP1 were analyzed by immunoblotting using anti-GFP antibody. Equal loading of lysates and GST-Plk1 (a.a. 356–603) is indicated by coomassie staining. The lower graph indicates quantification of the 53BP1 signal on the Western blot. Signal was corrected for local background and input levels were set to 100%. (G) U2OS cells were left untreated of treated with nocodazole for 16 h. Nocodazole-treated mitotic cells were isolated by shake-off and, if indicated, subsequently treated with the Cdk1-inhibitor roscovitine for 30 min. Cell lysates were analyzed using anti-53BP1, anti-phospho-S376-53BP1, or anti-β-actin antibodies.

The NetworKIN algorithm, in addition to predicting 53BP1 as a substrate for Cdk1, also predicted putative Plk1 phosphorylation and PBD binding-site(s) in 53BP1 (Figure 2B). To investigate the functional roles of these sites, we immunoprecipitated endogenous 53BP1 from interphase or mitotic U2OS cells and examined the immunoprecipitates for co-association of Plk1 (Figure 4C). Whereas 53BP1 and Plk1 did not co-immunoprecipitate during interphase, a significant amount of Plk1 interacted with 53BP1 during mitosis (Figure 4C and Figure S1A). In addition to binding to 53BP1, Plk1 was able to efficiently phosphorylate 53BP1 in vitro (Figure 4D). To further identify the site(s) in 53BP1 that interact with Plk1, mutational analysis was performed (a selection of phosphorylation site mutants is indicated in Figure 4A,B). Lysates of interphase or mitotic U2OS cells stably expressing wt or mutant forms of GFP-tagged murine-53BP1 were incubated with the recombinant GST-tagged PBD from Plk1 (residues 363–562). Mitotic forms of 53BP1 show reduced migration on low percentage SDS-page gels, resulting in multiple bands in mitotic lysates from cells expressing endogenous as well as GFP-tagged 53BP1 (Figure 4E). As expected, wt-GFP-m53BP1 was efficiently pulled down by GST-PBD from mitotic lysates, but not from interphase lysates (Figure 4E). Like wt-GFP-m53BP1, both the GFP-m53BP1-1103A and mGFP-m53BP1-1620A mutants (corresponding to residues 1114 and 1635 in human 53BP1) efficiently bound to the Plk1 PBD (Figure 4F and unpublished data). A third predicted PBD binding site within 53BP1 (S380) resides in a cluster of potential PBD binding sites (some of which have not yet been shown to be phosphorylated in vivo). A 53BP1 mutant lacking this cluster of potential PBD binding sites (GFP-m53BP1 Δ196–439) did not interact with the PBD of Plk1 (Figure 4F). Importantly, the single highly conserved and predicted PBD-binding site that is found phosphorylated in vivo, S380 (corresponding to the S376 in murine 53BP1), appeared to be essential for the mitotic interaction between 53BP1 and Plk1, as the GFP-m53BP1-376A mutant could not be precipitated from mitotic lysates with recombinant PBD (Figure 4F). Furthermore, re-analysis of in vivo phosphorylation sites from mitotic Plk1 PBD pull-downs revealed the presence of phospho-S380 peptides from endogenous 53BP1 [44]. Combined, these results indicate that S380 is a critical site amongst the predicted CDK1/2 sites that is required for stable binding to Plk1. Although mass-spectrometry-based phospho-proteomics previously identified S380 as an in vivo phosphorylation site [41],[44], the dynamics of S380 phosphorylation during different phases of the cell cycle are unclear. In agreement with a model in which S380 is phosphorylated during mitosis, we could only observe S380 phosphorylation of 53BP1 in mitotically arrested cells, but not in interphase cells using a phospho-specific antibody raised against this site (Figure 4G). Furthermore, detailed analysis of phosphorylation during the cell cycle revealed intense phosphorylation of S380 when synchronized cells entered mitosis (Figure 3D), consistent with this site being a Cdk1 target. Finally, treatment of mitotic cells with the Cdk1 inhibitor roscovitine eliminated S380 phospho-reactivity (Figure 4G).

### 53BP1 Is Not Involved in Normal Mitotic Progression

**Figure 5:**
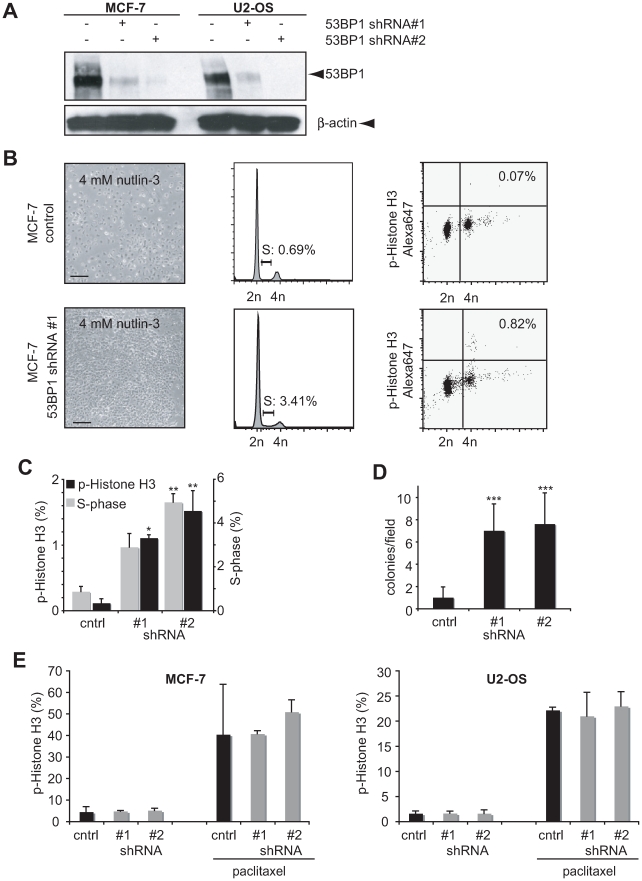
Cell cycle analysis of 53BP1-depleted cells.

Although the identification of mitotic phosphorylation sites in DNA damage checkpoint proteins can elucidate potential feedback targets within the checkpoint networks, it is conceivable that mitotically phosphorylated checkpoint proteins could also possess alternative cellular functions. Mitotic phosphorylation of such proteins could, for example, be important for the regulation of normal mitotic progression, rather than facilitating feedback control during an exogenous G_2_ DNA damage checkpoint response. To investigate a possible role for 53BP1 during an unperturbed mitosis, we stably infected U2OS or MCF7 cell lines with 53BP1 RNAi hairpins and examined these cells for possible defects in mitotic progression (Figure 5). We used two independent hairpins that significantly decreased 53BP1 levels in both U2OS and MCF7 cell lines (Figure 5A). To select for a functional 53BP1 knockdown, MCF7 cell lines were treated with the MDM2 inhibitor Nutlin-3 [60]. Nutlin-3 treatment leads to a cell cycle arrest that depends on p53 as well as 53BP1 [60],[61]. As expected and reported previously, knockdown of 53BP1 significantly increased mitotic indices, the number of cells in S-phase, as well as the size and number of proliferating colonies following Nutlin-3 treatment (Figure 5B,C,D and unpublished data) in the 53BP1 knockdown cells. Although the increases in M- and S-phase content after Nutlin treatment in 53BP1 knockdown cells is minor, the increase in colony formation suggests that this effect is meaningful. A functional 53BP1 knockdown was also evidenced by a small but highly reproducible increase in mitotic content after low dose (2 and 3 Gy) ionizing radiation (unpublished data). In contrast, no differences in mitotic indices were observed in the untreated cell population, indicating that loss of 53BP1 does not interfere with normal mitotic progression. Likewise, paclitaxel treatment resulted in similar increases in the percentages of mitotic cells in control and 53BP1-depleted lines, suggesting that 53BP1 is not required for normal functioning of the spindle assembly checkpoint (Figure 5E).

### Mitotic Phosphorylation of 53BP1 Contributes to DNA Damage Checkpoint Exit

**Figure 6:**
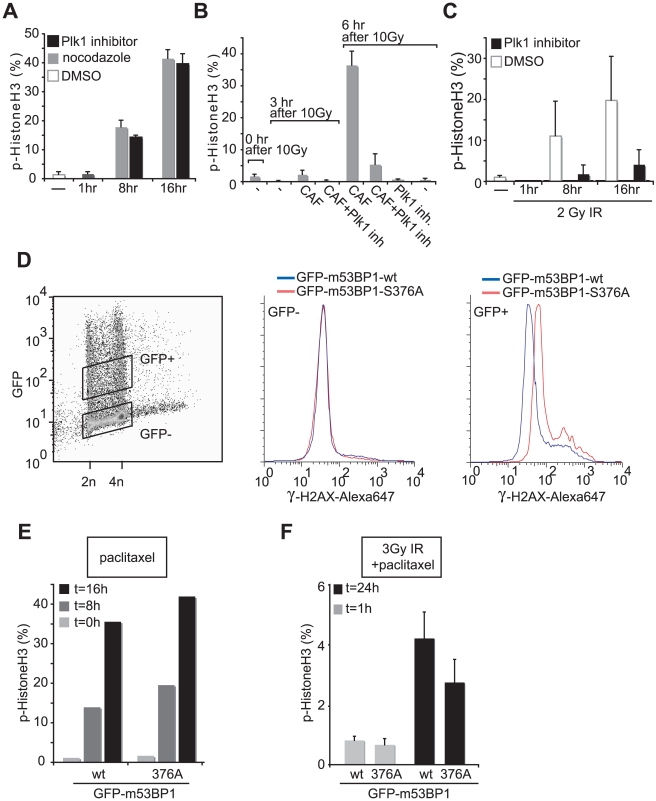
Phosphorylation of 53BP1 controls DNA damage checkpoint release. (A) U2OS cells were treated for indicated time periods with DMSO, paclitaxel, or the Plk1 inhibitor BI 2536. Cells were stained using anti-phospho-Histone H3 and analyzed by FACS. Mean values and SEM from three experiments are indicated. (B) U2OS cells were left untreated or treated with 10 Gy ionizing irradiation (IR). Twelve hours after irradiation (indicated as t = 0 h), cells were left untreated or incubated with or without caffeine in the absence or presence of the Plk1 inhibitor BI 2536 for 3 or 6 h in the presence of paclitaxel to visualize cumulative mitotic entry. Phospho-Histone H3 content was measured by FACS. (C) U2OS cells were left untreated or treated with 2 Gy ionizing irradiation (IR). Thirty minutes after irradiation, cells were incubated with paclitaxel in the absence (white bars) or presence of Plk1 inhibitor (black bars). Cells were harvested at 1, 8, and 16 h after paclitaxel addition, and phospho-Histone H3 content was determined by flow cytometry. (D) U2OS were infected with *wt*- or S376A-EGFP-m53BP1 and, 48 h later, irradiated with 3Gy IR. Cells were harvested 12 h later, stained with anti-γ-H2AX, and analyzed by FACS. Blue lines indicate γ-H2AX levels from cells infected with retroviruses encoding *wt*-EGFP-m53BP1 while red lines indicate γ-H2AX levels from cells infected with retroviruses encoding S376A-EGFP-m53BP1. GFP-positive (infected) cells and GFP-negative (uninfected) cells are plotted separately. (E) U2OS cells were infected with retroviruses encoding *wt*-EGFP-m53BP1 or S376A-EGFP-m53BP1 and treated with paclitaxel for indicated time periods. Percentages of phospho-Histone H3-positivity within the GFP-positive cell population were analyzed by FACS. (F) U2OS cells were infected with retroviruses encoding *wt*-EGFP-m53BP1 or the S376A-EGFP-m53BP1 mutant and, 48 h later, irradiated with 3 Gy. Thirty minutes after irradiation, paclitaxel was added and the percentages of phospho-Histone H3-positive cells within the GFP-expressing cell populations were determined by flow cytometry at the indicated times. Mean values and SEM from three independent experiments are shown.

Our finding that 53BP1 is not involved in spindle checkpoint functioning allowed us to use microtubule poisons to trap cells in mitosis after checkpoint escape, even in cells with modulated 53BP1 expression levels. In these experiments, if the observed mitotic phosphorylation of 53BP1 is important for attenuating the DNA damage checkpoint, one would expect to observe altered kinetics of G2-M transition when phosphorylation site mutants of GFP-m53BP1 are expressed, especially after cells are treated with genotoxic compounds. To first assess how phosphorylation by mitotic kinases alters the function of checkpoint components such as 53BP1, we utilized genetic and chemical inhibition of Plk1. Previously, a role for Plk1 in checkpoint silencing was identified by using siRNA technology [32]–[36]. Although clear differences in cell cycle reentry were observed after silencing Plk1 expression, a limitation of these RNAi experiments is that they cannot distinguish between a requirement for the mere presence of Plk1 in checkpoint recovery or for the enzymatic activity of Plk1 during this process. We therefore wished to confirm these results using the temporally controlled chemical inhibition of Plk1 [62]. As previously reported, chemical inhibition of Plk1 using BI-2536 led to spindle checkpoint activation and a concomitant mitotic arrest [63] with kinetics similar to those seen in nocodazole- or paclitaxel-treated cells (Figure 6A and unpublished data). Moreover, when the G_2_ DNA damage checkpoint was activated in U2OS cells by γ-irradiation, and the checkpoint then abrogated by treatment of the damaged cells with the ATM/ATR inhibitor caffeine, the cells rapidly entered mitosis, where they could be trapped in the presence of paclitaxel (Figure 6B). In contrast, cells treated with the Plk1 inhibitor were unable to enter mitosis and remained in G_2_, clearly indicating that Plk1 kinase activity, rather than physical presence of Plk1 per se, is required for cell cycle reentry after a DNA damage checkpoint arrest when the upstream checkpoint signaling pathways are silenced with caffeine. This effect does not appear to result from DNA damage induced by Plk1 inhibition, as was previously suggested [64], since Plk1 inhibition did not initiate DNA damage-induced foci (Figure S1C).

In addition to caffeine-induced checkpoint abrogation, we could show that Plk1 activity was equally important for spontaneous checkpoint recovery (Figure 6C). In response to low dose IR (2 Gy), U2OS cells delay cell cycle progression for up to 8 h, during which time cumulative mitotic entry is significantly lower (Figure 6C). When cells were treated with the Plk1 inhibitor following low-dose DNA damage checkpoint activation, similarly low mitotic indices were observed. However, unlike control cells in which the mitotic index had recovered to approximately 80% of the levels seen in unirradiated cells by 16 h after 2 Gy ionizing radiation, cells that were irradiated and treated with the synthetic Plk1 inhibitor maintained persistently low mitotic indices (Figure 6C). These results confirm a specific role for the kinase activity of Plk1 in spontaneous cell cycle reentry after a G2 DNA damage checkpoint arrest, as well as the requirement for Plk1 for normal mitotic progression beyond metaphase [31],[32],[34],[35],[65],[66].

Next, to explore whether the interaction of 53BP1 with Plk1 was important for the DNA damage recovery phenotype, we irradiated U2OS cells, expressing GFP-tagged wt-m53BP1 or a GFP-53BP1 mutant that was unable to bind Plk1 (Figure 6D), and monitored persistence of DNA damage checkpoint activity 24 h later by quantitatively measuring levels of H2AX phosphorylation by flow cytometry. As shown in Figure 6D, both the control untransfected cells and the cells expressing wt-53BP1 showed only background levels of γ-H2AX staining by this time after irradiation. In contrast, 24 h after irradiation cells expressing the Plk1-binding mutant GFP-m53BP1-S376A showed persistently increased γ-H2AX-positivity (Figure 6D). To assess the effects of such altered checkpoint activation on cell cycle progression, a parallel set of studies was performed in the absence (Figure 6E) or presence of low-dose IR (Figure 6F), and mitotic entry quantified by measuring phospho-Histone H3 staining in the presence of paclitaxel to trap all cells exiting G_2_ in mitosis. As shown in Figure 6E, in the absence of DNA damage cells, expressing the S376A-m53BP1 mutant showed no reduction in mitotic entry—if anything, the percentage of pH3-positive cells was slightly increased in m53BP1 mutant-expressing cells. In contrast, cells expressing S376A-m53BP1 were delayed in mitotic entry after irradiation with low-dose IR compared to either untransfected cells (unpublished data) or cells expressing wt-m53BP1 (Figure 6F), in agreement with the observed increase in checkpoint activity. These results strongly suggest that mitotic regulation of 53BP1 by Plk1 modulates DNA damage checkpoint activity to control checkpoint recovery.

It was previously suggested that 53BP1 functions as a molecular platform/scaffold for the efficient recruitment, phosphorylation, and activation of several checkpoint components including p53, BRCA1, and Chk2 [57],[67]–[70]. Chk2 is a Ser/Thr kinase that possesses an SQ/TQ-rich N-terminus, an N-terminal phosphopeptide-binding Forkhead-Associated (FHA) domain that is crucial for Chk2 activation, and a C-terminal kinase domain. Specifically, 53BP1 was shown to be required for Chk2 activation in response to DNA damage, as Chk2 activation was shown to be significantly impaired in 53BP1 null cells and in cells where 53BP1 was depleted by RNAi [57],[69],[70], particularly when exposed to low doses of IR [70], or when signaling through the MDC1 branch of the DNA damage signaling pathway is suppressed [69],[71],[72]. Interestingly, the inability of Chk2 to be activated during mitosis (Figure 1B,C) strongly correlates with the absence of 53BP1 from DNA damage–induced foci in irradiated mitotic cells (Figure 3C) and with the mitotic phosphorylation of 53BP1 on Ser-376 to generate a Plk1 PBD binding site. These data suggest that 53BP1 may function as a docking platform where Plk1 and Chk2 can bind and possibly interact.

### Plk1 Can Disable Chk2 by Phosphorylating the FHA Domain

**Figure 7:**
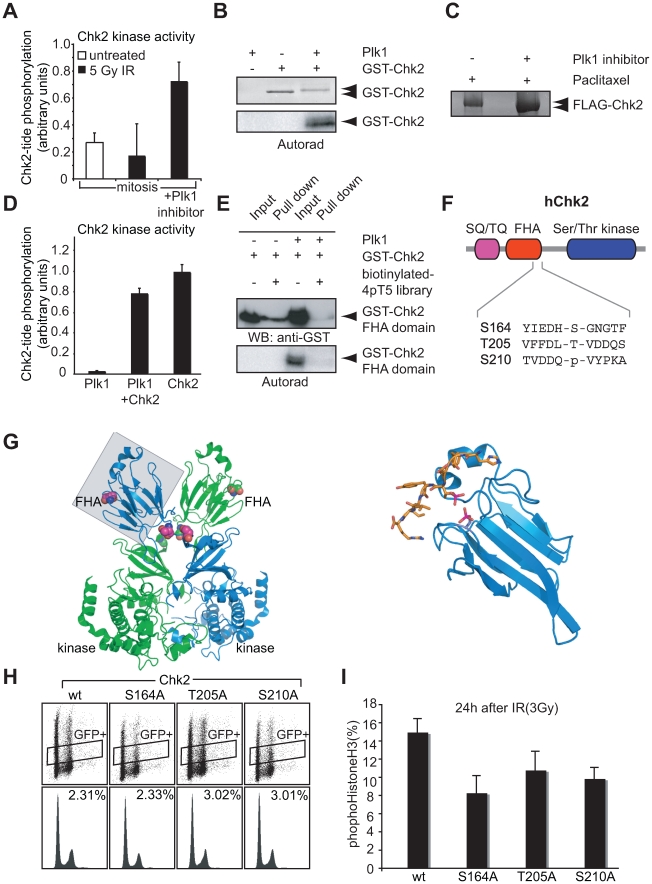
Inactivation of Chk2 by Plk1. (A) U2OS cells were treated with nocodazole in the absence or presence of the Plk1-inhibitor BI 2536 for 16 h. Mitotic cells were collected using gentle shake-off and subsequently irradiated (5Gy) as indicated. Chk2 was immunoprecipitated and kinase activity was assessed by incorporation of [^32^P]-γ-ATP into the optimal substrate peptide (“Chk2-tide”) in an in vitro kinase assay. Means and standard deviations from three independent experiments are shown. (B) A recombinant GST fusion of full-length Chk2 was incubated with Plk1 kinase domain in the presence of [^32^P]-γ-ATP. Samples were analyzed by SDS-PAGE and imaged by Coomassie staining (top) and autoradiography (bottom). (C) 293T cells were transfected with FLAG-Chk2. Twenty-four hours after transfection, cells were treated with paclitaxel in combination with DMSO or in combination with Plk1 for 8 h. FLAG-Chk2 was subsequently immunoprecipitated from lysates, analyzed by SDS page, and imaged by staining. (D) Following in vitro phosphorylation by Plk1 as in panel B, the kinase activity of Chk2 was measured by the incorporation of [^32^P]-γ-ATP into the optimal substrate peptide (“Chk2-tide”). A reaction containing Plk1 but lacking Chk2 is shown as a control. Data are normalized to the amount of Chk2tide phosphorylation observed for Chk2 alone. (E) The Chk2 FHA domain, prior to or following phosphorylation by Plk1 in the presence of [^32^P]-γ-ATP, was incubated with a biotinylated phosphothreonine peptide library bound to streptavidin beads. Input (10%) and bead-bound material was analyzed by SDS-PAGE and immunoblotting with anti-GST, or by autoradiography to assess phosphorylation state. (F) Schematic representation of human Chk2. Evolutionarily conserved phosphorylation sites in the FHA domain that match the optimal Plk1 consensus motif and were identified following in vitro phosphorylation by mass spectrometry are indicated. (G) A structural basis for Plk1-mediated inactivation of Chk2. Left panel: The near full-length Chk2 dimer is shown in ribbons representation, with monomers colored green and cyan. Residues phosphorylated by Plk1 are shown in space filling representation. Right panel: The isolated Chk2 FHA domain∶phosphopeptide complex, shown in the same orientation as the boxed region in the left panel. The phospho-threonine peptide ligand and the modeled side chain of phospho-Ser-164 are shown in stick representation with phosphates colored purple. (H) U2OS cells were transfected with the indicated pIRES2-Chk2 plasmids co-expressing GFP. Cells were fixed and stained with PI and an anti-phospho-HistoneH3 antibody. GFP-positive cells were gated and the corresponding DNA profiles and percentages of phospho-HistoneH3-positive cells are indicated on the lower panels. (I) U2OS cells were treated as in panel H. Cells were left untreated for 48 h or irradiated (3Gy) and subsequently treated with paclitaxel for 24 h. Percentage of GFP-positive cells that are phosphoHistoneH3 positive at 24 h after irradiation are shown. Averages and standard errors of two experiments are shown.

To test the hypothesis that Plk1 kinase activity could inhibit Chk2 as part of the mechanism of checkpoint inactivation, we first examined whether the activity of Plk1 could be responsible for the inability of DNA damage to activate Chk2 during mitosis (Figure 1B,C). In these experiments, U2OS cells were treated with nocodazole in the absence or presence of the Plk1 inhibitor BI 2536, and mitotic cells then isolated and irradiated with 5 Gy of ionizing radiation. Chk2 activity was measured 1 h after irradiation using an immunoprecipitation/in vitro kinase assay (Figure 7A). No increase in Chk2 kinase activity was observed in the irradiated mitotic cells compared to the unirradiated mitotic cells, as expected. If the mitotic cells were treated with the Plk1 inhibitor, however, a marked elevation of Chk2 kinase activity was seen after DNA damage, consistent with a model where Plk1 kinase activity suppresses Chk2 activity during mitosis. We next examined whether Chk2 could be a direct substrate of Plk1. As shown in Figure 7B, incubation of full-length Chk2 with Plk1 in the presence of [^32^P]-γ-ATP resulted in significant Chk2 phosphorylation, as visualized by ^32^P incorporation and a clear phosphorylation-induced mobility shift (Figure 7B). In order to examine whether these effects could be recapitulated in vivo during checkpoint recovery, we irradiated U2OS cells expressing FLAG-tagged Chk2 in the absence or presence of Plk1 inhibitor (Figure 7C). Following checkpoint inactivation using caffeine, FLAG-Chk2 was immunoprecipitated and analyzed by SDS-PAGE. Cells treated with the Plk1 inhibitor showed a markedly faster migrating form of Chk2, confirming that the Plk1-dependent modification that was observed in vitro also occurs in vivo. Surprisingly, in vitro phosphorylation of Chk2 by Plk1 had only a minor effect on the ability of the Chk2 kinase domain to phosphorylate an optimal peptide substrate (Figure 7D). In marked contrast, in vitro phosphorylation of the FHA domain of Chk2 by Plk1 completely abrogated the ability of the FHA domain to bind to its phosphopeptide ligands (Figure 7E). Since the FHA domain is known to be critical for DNA damage–induced phosphorylation, oligomerization, and activation of Chk2 in vivo [73]–[76], our results indicate that loss of Chk2 activation and function in cells during both mitosis and recovery from a DNA damage checkpoint likely involves contributions from both Plk1 binding to 53BP1 and direct phosphorylation-induced inactivation of the Chk2 FHA domain. To further examine this, the Plk1 phosphorylation sites within the FHA domain of Chk2 were mapped using nano-liquid chromatography and mass spectrometry (Figure 7F and Figure S2A–C), revealing three sites, Ser-164, Thr-205, and Ser-210, that are both evolutionarily conserved and match the optimal phosphorylation motif for Plk1 ([77]; Alexander and Yaffe, manuscript in preparation). Mapping of these sites onto the X-ray crystal structures of the Chk2 FHA∶phosphopeptide complex [78] and the recently solved structure of the near-full-length Chk2 dimer (Figure 7G) [79] reveals that one of these sites, Ser-164, is in close proximity to the phosphopeptide-binding site, with its phosphorylation likely to disrupt ligand binding through electrostatic repulsion of the ligand phosphothreonine residue (Figure 7G right panel). Both Thr-205 and Ser-210 lie at the interface between the two monomers in the dimeric Chk2 structure that is believed to represent the early stages in the Chk2 activation process [79]. Phosphorylation of these residues would be expected to disrupt both the dimeric FHA∶FHA domain interaction as well as the interaction between the FHA domain of one monomer with the kinase-FHA linker of the other (Figure 7G left panel). It is not technically possible to directly assay Plk1-dependent alterations in phosphopeptide-binding capacity of the Chk2 FHA domain within cells expressing wild-type or mutant 53BP1. Therefore, to determine if phosphorylation of the FHA domain by Plk1 contributes to the observed Plk1 dependence of checkpoint silencing, we tested whether mutation of the identified phosphorylation sites affected the ability of cells to recover from a DNA damage checkpoint arrest. In these experiments, cells were transfected with wild-type or mutant forms of Chk2 in which each of the phosphorylation sites was replaced by Ala, along with an IRES-driven GFP (Figure 7H). Expression of wild-type or mutant forms of Chk2 did not result in altered cell cycle distributions under untreated conditions (Figure 7H). In marked contrast, mutation of Ser-164, Thr-205, or Ser-210 to a non-phosphorylatable residue was found to clearly impair checkpoint recovery, as judged by a significant decrease in cumulative mitotic entry at 24 h after irradiation (Figure 7I), with mutation of Ser-164 showing the greatest effect. These results show that Chk2 phosphorylation by Plk1 inhibits the function of the FHA domain and that these phosphorylation events contribute to inactivation of the DNA damage checkpoint during mitosis and checkpoint recovery.

## Discussion

In response to genotoxic injury, cells activate a network of DNA damage signaling pathways involving the upstream serine/threonine kinases ATM and ATR and the downstream kinases Chk1, Chk2, and MK2 to induce G_1_, S, and G_2_ cell cycle arrest, recruit repair machinery to the sites of damage, and target irreversibly damaged cells for apoptosis [4],[80]. ATR and its downstream effector kinase Chk1 are essential genes that respond primarily to single-strand DNA lesions and bulky base modifications. In contrast, the ATM-Chk2 signaling pathway, which is activated by DSBs (considered to be the most lethal type of DNA damage), is composed of nonessential genes. Their importance, however, is highlighted by the observation that interference with ATM and Chk2 function severely impairs the checkpoint response to IR and other DSB-inducing lesions, and mutation of the genes encoding for ATM and Chk2 results in the cancer-prone Ataxia-Telangiectasia syndrome, and familial breast and prostate cancer, respectively [81]–[87]. Following DNA repair, cells must extinguish the DNA damage signal to allow the cells to reenter the cell cycle, but the mechanisms through which this occurs, particularly with respect to the ATM-Chk2 pathway, are poorly understood.

Since DNA damage checkpoints respond to as little as a single DNA DSB in model systems [25],[26], it has long been assumed that human cells also maintain the G_2_/M checkpoint until all of the breaks are repaired. Recent evidence, however, shows that the G_2_ checkpoint in immortalized human cells in culture displays a defined threshold of approximately 10–20 DSBs [23]. Limited checkpoint control was not only apparent in response to IR doses that cause very few DNA DSBs, cells that had also repaired more extensive amounts of DNA damage also showed checkpoint release when fewer than 10–20 DSBs were left unrepaired [23]. Although the fate of cells that continue proliferating in the presence of unrepaired DNA breaks is unclear, and the identity of the rate-limiting DNA damage checkpoint components has yet to be uncovered, a picture is emerging in which certain cues are capable of overriding the DNA damage checkpoint machinery. G_2_ checkpoint escape in the presence of unrepaired DNA damage may be particularly common during the evolution of cancer cells [2],[4],[5],[8],[9], reinforcing the need to better understand this process in molecular detail.

Recently, a pathway comprising Aurora A, Bora, and Plk1 was shown to control inactivation of the G_2_ DNA damage machinery [65],[88]. Although several targets of Plk1 within or downstream from the ATR-Chk1 pathway that are involved in DNA damage checkpoint silencing have been described, no target within the ATM-Chk2 pathway has been identified thus far [32]–[36]. Here, we have used a combined bioinformatics and biochemical approach to identify targets of mitotic kinases within the DNA damage checkpoint. We show that the 53BP1 checkpoint protein interacts with Plk1 and is phosphorylated by Cdk1/Cyclin B and Plk1. In addition, we show that expression of a 53BP1 mutant that is unable to interact with Plk1 prevents proper checkpoint release. 53BP1 was previously identified as a non-enzymatic DNA damage checkpoint mediator protein that is recruited to sites of DNA damage through protein-protein interactions, oligomerization, and binding to methylated histones [89]–[94]. Although the recruitment of 53BP1 to sites of DNA damage has been studied intensively, the exact functions of 53BP1 are only beginning to emerge. 53BP1 was recently shown to regulate DNA repair as a component of the NHEJ network [18],[19],[95]. In addition, 53BP1 regulates checkpoint responses by interacting with a range of downstream checkpoint components, including Chk2 and p53 [57],[61],[69],[70].

**Figure 8:**
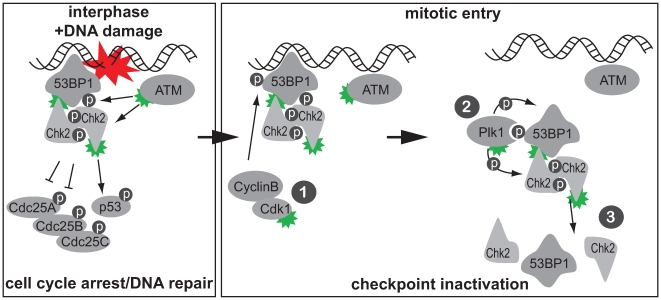
A model for mitotic checkpoint inactivation. One model for checkpoint inactivation at the G2-M transition. Left panel: DNA lesions promote the formation of protein complexes, including 53BP1 and Chk2, that mediate checkpoint function and promote DNA repair. Green symbols indicate active kinases. Right panel: (1) To terminate the ATM-Chk2 branch of the G2/M checkpoint, CyclinB/Cdk1 phosphorylates DNA damage signaling proteins, including 53BP1. (2) Cdk1 phosphorylation of 53BP1 creates a Plk1 PBD docking site, leading to Plk1 recruitment, phosphorylation of checkpoint components, and inactivation of the Chk2 FHA domain. (3) These combined phosphorylation events by mitotic kinases drive cell cycle reentry and prevent further DNA damage checkpoint activation during mitosis.

Our results strengthen a role for 53BP1 as a checkpoint regulator and indicate that 53BP1 functions as a binding platform for Plk1 during the checkpoint recovery process. This suggests a model in which 53BP1 might mediate a direct interaction between Plk1 and the 53BP1-binding protein Chk2. We suggest that mitotic Cdk1 phosphorylation of 53BP1 and subsequent interaction of Plk1 and 53BP1 may function to bring Plk1 and the 53BP1-interacting protein Chk2 in close proximity (Figure 8, step 1). Subsequent direct phosphorylation of Chk2 by Plk1 (Figure 8, step 2) leads to impaired Chk2 phosphopeptide-binding ability by its FHA domain, which is required for continued Chk2 activation and function in cell cycle arrest (Figure 8, step 3). Our results fit well with previous observations in fission yeast in which a prolonged DNA damage–induced checkpoint arrest was observed when Cdk phosphorylation site mutants of the 53BP1 homologue Crb2 were expressed [96]. The budding yeast Polo-like kinase homologue Cdc5 has also been shown to be required for DNA damage checkpoint silencing in the presence of persisting DSBs [29]. Moreover, *S. cerevisiae* cells lacking a wt-CDC5 allele were unable to silence the activity of the Chk2 homologue Rad53 [97], indicating that, directly or indirectly, Polo-like kinase may regulate Chk2 function in that organism. The budding yeast 53BP1/Mdc1 homologue Rad9 has been shown to regulate checkpoint responses to DNA damage. Similar to 53BP1, Rad9 is activated by the ATM/ATR homologues Tel1/Mec1 and associates with the Chk2 homologue Rad53 [98]–[100]. A role for Rad9 as a target for feedback control to silence checkpoint functioning, however, has not yet been shown.

In addition to constituting a mechanism for silencing an activated DNA damage checkpoint, the Cdk1-Plk1-53BP1 feedback loop may be a more general means to prevent activation of the DNA damage checkpoint during mitosis. If a DNA damage–induced G2-like checkpoint were to become fully functional during mitosis, DNA lesions encountered during mitotic progression could result in inactivation of Cdk1/Cyclin B and result in forced mitotic exit. Such an event would cause the accumulation of 4N-DNA containing interphase cells, which were recently shown to have increased tumorigenic potential [101],[102]. Hence it can be expected that cellular mechanisms exist to prevent inappropriate Cdk1 inactivation during mitosis. Indeed, DNA damage during mitosis had previously been shown to be unable to delay mitotic progression or alter Cdk1 activity during mitosis [39],[40]. Our observation that Chk2 cannot be catalytically activated during mitosis by IR further strengthens this notion.

Immortalized proliferating cells are believed to have increased replication stress and elevated levels of associated DNA damage. The DNA damage checkpoint, therefore, was shown to form a barrier against malignant transformation [8],[9]. Feedback mechanisms, in which mitotic kinases can silence DNA damage checkpoints, may thus explain why Plk1 and Aurora A are frequently overexpressed in cancers and may form a rationale for including inhibitors of such mitotic kinases during cancer treatment [103]–[105].

## Materials and Methods

### Bioinformatics Analysis of Conserved Phosphorylation Sites in the DNA Damage Signaling Network

A total of 244 in vivo mapped phosphorylation sites on 33 human DDR-related proteins were manually collected from Phospho.ELM [42], Phosphosite [43], and a phospho-proteomic study of Polo Box substrates [44]. The conservation level of these phosphorylation sites was measured by aligning predicted orthologues of these proteins in 11 species from the high-coverage vertebrate branch of Ensembl (release 46; [106]) with the human seed sequences in which the sites had been mapped by mass spectrometry and other means. Genomes used in the analysis included Homo sapiens (human), Bos taurus (cow), Canis familiaris (dog), Danio rerio (zebrafish), Gallus gallus (chicken), Macaca mulatta (rhesus monkey), Mus musculus (house mouse), Ornithorhynchus anatinus (platypus), Rattus norvegicus (Norway rat), Tetraodon nigroviridis (fresh water pufferfish), and Xenopus tropicalis (western clawed frog).

Ortholog mapping was performed as follows: initially the human DDR sequences and phosphorylation sites were mapped to their corresponding Ensembl gene entries by sequence comparison. Next, genes orthologous to the DDR genes were retrieved from Ensembl. Orthologous relationships between human and other vertebrate genes in Ensembl were inferred from phylogenetic trees constructed from multiple sequence alignment of CDS sequences [106]. A detailed description of Ensembl ortholog detection pipeline in release 46 is available at http://aug2007.archive.ensembl.org/info/data/compara/homology_method.html. Finally, each DDR protein sequence and all spliced variants of its orthologous genes across the 11 species were aligned using multiple-sequence alignment program MAFFT (v6.240, E-INS-i option with default parameters) [107].

Cross-species conservation of the human phosphorylation sites was then computed by evaluating the average number of amino acid substitutions within a −5 to +5 residue window of the modified residue (S, T, or Y) across the 11 vertebrate genomes from the sequence alignments using Perl scripting. S→T and T→S transitions of the central phosphoresidue were permitted, but S/T→Y transitions were not. The conservation level for each phosphorylation site is reported as the average across the 11 genomes, as a percentage of conserved residues within the 11-mer window, if the corresponding S/T is conserved.

Information about which of the 244 in vivo mapped phosphorylation sites were phosphorylated by the specific kinases ATM/ATR, Cdk1/2, Chk1/2, and Plk1 was collected from Phospho.ELM [42] and Phosphosite [43], along with whether phosphorylation at that site was known to create a binding site for the PBD of Plk1 [44]. In cases where multiple kinases are known to phosphorylate a single site, all of this information was retained and displayed. For sites where the upstream kinase was not experimentally known, we predicted the likely kinase responsible for phosphorylation at that site by computational analysis using the programs NetworKIN [45],[47] and NetPhorest [46].

### Antibodies, Plasmids, and Reagents

Rabbit anti-53BP1 (304-A1) was from Novus Biologicals. Mouse anti-γ-H2AX (pS139, #05-636), rabbit anti-HistoneH3 pS10 (#06570), rabbit anti-Chk2 (#2662), rabbit anti-Chk2-pT68 (#2661), rabbit anti-53BP1-pS1778 (#2675), mouse anti-MPM2 (#05-368), and rabbit anti-Plk1 (#06-831) were purchased from Upstate. An additional rabbit anti-Chk2 antibody (#BL1432) was purchased from Bethyl Laboratories. Rabbit anti-Plk1 for immunoprecipitation was a kind gift from Dr. René Medema. Mouse anti-β-actin (A5441) was from Sigma. Mouse anti-Cyclin B1 (GNS1, sc-245), rabbit anti-GFP (sc-8334), and rabbit non-specific IgG (sc-2025) were from Santa Cruz Biotechnology. Mouse anti-GFP (clones 7.1 and 13.1) was from Roche. Rabbit anti-p-S380-53BP1 phospho-specific antibody was raised against peptide Pro-Phe-Iso-Val-Pro-Ser-pSer-Pro-Thr-Glu-Gln-Glu-Gly-Arg-Tyr and purified by Cell Signaling Technologies. Radiolabelled [^32^P]-γ-ATP (3,000 Ci/mmol) was purchased from Amersham/GE Healthcare. Plk1 inhibitor (BI 2536) was synthesized following the procedure described by Munzert et al. [108]. All other reagents and chemicals were from Sigma unless otherwise indicated.

The pEGFP-m53BP1 expressing murine GFP-Tagged 53BP1 was kindly provided by Dr. Yasuhisa Adachi. The Nhe1-Apa1 fragment of pEGFP-m53BP1 was cloned in the retroviral plasmid pLNCX2 (Clontech) containing a synthetic linker to generate pLNCX2-GFP-m53BP1. PCR-based mutagenesis was used to create pLNCX2-GFP-m53BP1-317A, m53BP1-330A, m53BP1-376A, m53BP1-922A, m53BP1-1103A, and m53BP1-1620A. All plasmid constructs were verified by automated sequencing. pLNCX2-GFP-m53BP1Δ196–439 was created by a nested PCR on two m53BP1 PCR fragments surrounding the deletion. The resulting 53BP1 fragment containing the deletion was used to replace wt-m53BP1 in pLNCX2-GFP-m53BP1. Full-length human Chk2 was cloned from pGEX6P2-Chk2 and subcloned into the Nhe1-EcoR1 sites of pIRES2-GFP (Clontech). Serine/Threonine to Alanine mutations at positions 164, 168, 205, and 210 were obtained by side directed mutagenesis and validated using automated sequencing. Full-length FLAG-tagged Chk2 was a kind gift from Dr. Domenico Delia. VSV-G pseudotyped retroviruses were prepared according to standard techniques. In brief, HEK293T packaging cells were transfected with the pLNCX-2 and the packaging plasmids pMDg/p and pMDg in a 4∶3∶1 ratio. Virus-containing supernatant was harvested at 24 and 48 h after transfection, filtered through a 0.45 µM syringe filter, and used to infect U2OS osteosarcoma target cells. A plasmid encoding the PBD of Plk1 (aa. 326–603) fused to GST was described previously [50].

### Cell Culture

U2OS osteosarcoma cells were maintained in Dulbecco's Modified Eagle medium, supplemented with 10% fetal calf serum, 100 units/ml penicillin, and 100 µg/ml streptomycin. To obtain mitotic cell populations, cells were incubated with paclitaxel (1 µg/ml) or nocodazole (250 ng/ml, Sigma). Where indicated, cells were harvested by mitotic shake-off. Where indicated, DNA damage was induced using a gamma-cell 40 irradiator equipped with a ^137^Cesium source for indicated doses. Alternatively, cells were incubated with doxorubicin (0.5 µM) for 1 h.

### RNA Interference

Human breast cancer cell line MCF7 or human osteosarcoma U2OS cells were retrovirally infected with control pRetrosuper or pRetrosuper-53BP1 (53BP1-targeting sequence #1, 5′-GATACTGCCTCATCACAGT-3′; 53BP1-targeting sequence #2 5′-GAACGAGGAGACGGTAATA-3′) for three consecutive 12 h periods [61]. Infected cells were selected with 2 µg/ml puromycin. pRS-53BP1-infected MCF7 cells were subsequently treated with 4 µM nutlin-3 to select for cells with a functional 53BP1 knockdown [61]. The statistical analysis of colony numbers, S-phase content, and phospho-HistoneH3 content in control-infected or pRS-53BP1-infected MCF-7 cells was done using the unpaired *t* test. Two-tailed *p* values were calculated using GraphPath software.

### Protein Purification

The Plk1 kinase domain (residues 38–346) was made as a His_6_-tagged construct in *Escherichia coli* (*E. coli*) Rosetta cells (Novagen) and purified by Ni-NTA chromatography followed by gel filtration on a Superose-12 column. Recombinant full-length GST-Chk2 and a GST-Chk2 FHA domain (amino acids 1–219) fusion were expressed and purified from *E. coli*. In brief, full-length Chk2 was cloned into pGEX-6P1 (GE Healthcare) and transformed into BL21 (DE3) cells. Cells were grown at 37°C to an OD600 of 0.6 and the culture temperature was reduced to 18°C for 30 min before a final concentration of 0.3 mM IPTG was added for overnight expression. Cells were pelleted and washed with MTPBS (16 mM Na_2_HPO4, 4 mM NaH_2_PO4, 150 mM NaCl, pH 7.3) and lysed by sonication in the same buffer with the addition of benzonase. The lysate was clarified by centrifugation, and the GST-Chk2 fusion protein was captured on glutathione 4B resin. After washing with 30 column volumes of phosphate-buffered saline (PBS), Chk2 was cleaved off the GST tag on the resin with 3C protease at 4°C overnight. The eluted full-length Chk2 was further purified by anion exchange on a Resource Q column (GE Healthcare) equilibrated with 20 mM Tris pH 8.0, 50 mM NaCl, 0.5 mM TCEP, and developed with 20 mM Tris pH8, 1 M NaCl, and 0.5 mM TCEP. Peak fractions containing full-length Chk2 were pooled and further purified with a Superdex S200 gel filtration column (GE Healthcare).

The GST-Chk2 FHA domain cloned into pGex-4T1 was transfected into BL21(DE3) cells, grown to an OD_600_ of 0.8, and induced with 1 mM IPTG at 37°C for 6 h. Cells were lysed in PBS containing 1 mM DTT and a mixture of protease inhibitors and disrupted by sonication. Benzonase (Novagen) was added at room temperature for 30 min and the lysate cleared via centrifugation. Roughly 500 µL of PBS-equilibrated GSH beads were added to the lysate and incubated at 4°C with rocking overnight. Non-bound material was aspirated off followed by 4×10 mL washes with PBS containing 0.2% NP-40 and 1 mM DTT, and the GST-Chk2 FHA domain eluted off the beads by incubation in 2.5 mL of elution buffer (20 mM HEPES pH 7.2, 40 mM glutathione, and 1 mM DTT; EB+G) at 4°C overnight. The purified GST-Chk2 FHA domain was dialyzed against elution buffer lacking 40 mM glutathione (EB) using a Slide-A-Lyzer (Pierce) dialysis cassette with a molecular weight cut-off of 6–8 kDa at 4°C with three buffer exchanges. Purity was assessed by SDS-PAGE and the protein concentration determined by absorbance at A_280_ using an extinction coefficient of 71,780 M^−1^ cm^−1^.

### Chk2 FHA Domain-Phospho-Peptide Binding Assay

Streptavidin beads (Pierce, 75 pmol/µL gel) were incubated with a 5-fold molar excess (relative to binding capacity) of a biotinylated phosphothreonine-oriented peptide library (B-4pT5; biotin-Gly-AHA-Gly-AHA-Met-Ala-X-X-X-X-pThr-X-X-X-X-X-Ala-Tyr-Lys-Lys-Lys-NH_2_, where X indicates a equimolar degenerate mixture of all amino acids except Cys, and pThr denotes phosphothreonine) in 50 mM Tris pH 7.5, 150 mM NaCl, 0.5% NP-40, and 1 mM EDTA for 30 min at 4°C. Beads were washed five times with the same buffer to remove unbound peptides and then 20 µL of the bead-immobilized library was incubated with 10 µg of GST-Chk2 FHA domain prior to or following in vitro phosphorylation of the FHA domain by Plk1 kinase in the presence of [^32^P]-γATP. After a 60 min incubation, the beads were washed five times with 50 mM Tris pH 7.5, 150 mM NaCl, 0.5% NP-40, and 1 mM EDTA. Bead-bound protein was released by addition of SDS sample buffer with heating to 95°C and resolved by SDS-PAGE on 10% denaturing gels. Gels were analyzed by autoradiography using a phosphorscreen and a Typhoon variable mode imager (GE Healthcare, or transferred to PVDF membrane and immunoblotted using an HRP-conjugated anti-GST antibody to visualize binding of the GST-FHA domain).

### Immunofluorescence

U2OS cells were seeded on glass cover slips and treated as indicated. After treatment, cells were fixed in 3.8% formaldehyde in PBS for 15 min at room temperature. Subsequently, cells were permeabilized with 0.1% TritonX100 in PBS for 5 min. After extensive washing, cells were blocked and stained in PBS-0.05% Tween20 and mounted on slides. Images were acquired on a Zeiss Axioplan-2 inverted microscope, equipped with a Hamamatsu Orca-ER digital camera using OpenLab software.

### Immunoprecipitation, In Vitro Phosphorylation, and Kinase Assays

After the indicated treatments, U2OS cells were lysed in lysis buffer (1% TritonX-100, 50 mM Tris-HCl (pH 7.5), 150 mM NaCl, 50 mM beta-glycerophosphate, 10 mM sodium pyrophosphate, 30 mM NaF, 1 mM benzamidine, 2 mM EGTA, 100 µM NaVO4, 1 mM dithiothreitol (DTT), 1 mM phenylmethylsulfonyl fluoride, 10 µg/ml aprotinin, 10 µg/ml leupeptin, 1 µg/ml pepstatin, and 1 µg/ml microcystin-LR) for 15 min at 4°C and cleared by high speed centrifugation. Protein concentrations were measured using the bicinchoninic acid assay (Pierce). 53BP1 was immunoprecipitated from 500 µg of clarified cell lysate using 3 µg of anti-53BP1 antibody and 50 µls of Protein-A-conjugated agarose beads (50% slurry) for 16 h. Immunoprecipitations were extensively washed and analyzed by SDS-Page and Western blotting. Alternatively, immunoprecipitations were subjected to in vitro phosphorylation by resuspension in kinase buffer (50 mM Tris-HCL pH7.5, 10 mM MgCl2, 1 mM EGTA, 2 mM DTT, 2 mM dithiothreitol, 0.01% BRIJ35, and 150 mM NaCl2), followed by addition of 25 µM unlabelled ATP, 10 µCi of [^32^P]-γ-ATP, and recombinant Cyclin A-Cdk2, Cyclin B-Cdk1, or Plk1 for 30 min. Kinase reactions were analyzed by SDS-page and autoradiography.

IP/kinase assays for Chk2 activity were performed as generally described [109] using lysates from either interphase cells or from mitotic cells generated by treating U2OS cells with 0.25 µg/ml nocodazole for 16 h followed by harvesting of the mitotitc non-adherent cells by gentle shaking. In brief, Protein A microtiter strips (Pierce) were coated overnight with 1.0 µg of anti-Chk2 antibody (Bethyl) or non-specific rabbit IgG per well and washed three times with blocking buffer (1% bovine serum albumin in 50 mM Tris-HCl (pH 7.5), 150 mM NaCl, 0.05% Triton X-100). Cell lysates (100 µgs) were placed in each antibody-coated well, incubated for 3 h, then washed twice with wash buffer (50 mM Tris-HCl (pH 7.5), 150 mM NaCl) and twice with kinase wash buffer (20 mM Tris-HCl (pH 7.5), 15 mM MgCl_2_, 5 mM beta-glycerophosphate, 1 mM EGTA, 0.2 mM Na_3_VO_4_, 0.2 mM DTT). Kinase reactions were performed in a total volume of 60 µl containing 20 mM Tris-HCl (pH 7.5), 15 mM MgCl_2_, 5 mM β-glycerophosphate, 1 mM EGTA, 0.2 mM Na_3_VO_4_, 0.2 mM DTT, 0.4 µM protein kinase A inhibitor, 4 µM protein kinase C inhibitor, 4 µM calmidazolium, 25 µM ATP, 10 µCi [^32^P]-γ-ATP, and 10 µM of Chk2tide substrate. Reactions were incubated for 60 min at 37°C, then terminated by addition of 60 µl of 20 mM EDTA. Forty µl of the terminated reaction mixture was transferred to a phosphocellulose filter plate (Millipore, Bedford, MA) containing 100 µl, 75 mM H_3_PO_4_, and mixed. The reaction contents were vacuum-filtered and washed five times with 75 mM H_3_PO_4_ and three times with 70% ethanol. Scintillation counting was performed using a Microbeta TRILUX luminescence counter.

In vitro phosphorylation of recombinant Chk2 or the Chk2 FHA domain by Plk1 was performed by incubating 3–10 µgs of the substrate proteins with Plk1 kinase domain in 50 mM Tris pH 7.5 containing 150 mM NaCl, 10 mM MgCl_2_, 100 µg/ml bovine serum albumen, 5 mM DTT, and 100–500 µM unlabelled ATP, in the presence or absence of 10–20 µCi [^32^P]-γ-ATP, for 60–120 min at 30°C. Kinase assays of recombinant Chk2 before or after Plk1 phosphorylation were performed in the above buffer containing 1 mM DTT, 20 µCi [^32^P]-γ-ATP, and 50 µM Chk2tide in a final reaction volume of 50 µl at 30°C for 60 min. Samples were quenched with an equal volume of 0.05% H_3_PO_4_, and 5 µl of the reaction spotted onto P81 paper, air dried, washed extensively with 0.05% H_3_PO_4_, and analyzed by scintillation counting.

Identification of Plk1 phosphorylation sites in the Chk2 FHA domain following in vitro phosphorylation was performed by separating the reaction products by SDS-PAGE. Gel slices containing Chk2 were excised, alkylated with iodoacetamide, and digested with trypsin. Peptides were resolved by nano-flow reversed phase liquid chromatography (Agilent 1100, Palo Alto, CA) and analyzed with a LTQ-Orbitrap equipped with a nanoelectrospray ionization source (Thermo, Bremen, Germany). Peptide and protein identification was analyzed using the Spectrum Mill MS Proteomics Workbench software (Agilent).

For the in vivo mobility shift analysis of Chk2, 293T cells were transfected with FLAG-tagged full-length hChk2. Twenty-four h after transfection, cells were treated with paclitaxel in combination with DMSO or in combination with Plk1 inhibitor for 8 h. Cell lysates were cleared by centrifugation and mixed with M2 FLAG resin for overnight immunoprecipitation. After washing, samples were analyzed by SDS-PAGE.

### Flow Cytometry

Cells were harvested with Trypsin/EDTA, washed with PBS, and subsequently fixed in ice-cold 70% ethanol for 4–16 h. After washing, cells were stained with anti-phospho-Histone H3 (1∶200) or anti-phospho-γ-H2AX (1∶100) in PBS-0.05% Tween20 and counterstained with Alexa647-conjugated secondary antibodies in PBS-0.05% Tween20. Cells were treated with Propidium Iodide/RNAse and analyzed on a Becton Dickinson FACScalibur using Cellquest software. A minimum of 10,000 events were counted.

## Supporting Information

Figure S1 (A) U2OS cells were left untreated or were treated with nocodazole for 16 h. Total cell lysates were immunoblotted using indicated antibodies (left panel). In parallel, cell lysates were used for anti-Plk1 or control (IgG) immunoprecipitations (right panel). Immunoprecipitations were washed extensively and immunoblotted for Plk1 and 53BP1. (B) Co-localization of 53BP1 with γH2AX in interphase but not mitosis. U2OS cells were left untreated or subjected to 3 Gy of ionizing radiation. Thirty minutes after irradiation, cells were fixed and immunostained using murine anti-γ-H2AX/anti-mouse-Alexa568 and rabbit anti-53BP1/anti-rabbit-Alexa488. Left panel: The number of nuclear foci per cell was counted from 30 interphase and 30 mitotic cells. Averages and standard error of the mean (SEM) are indicated. Middle panel: γ-H2AX foci from irradiated interphase and mitotic cells were analyzed for their co-localization with 53BP1 by visual inspection. One hundred and forty-six distinct γ-H2AX foci from 20 interphase cells and 76 discrete γ-H2AX foci from 30 mitotic cells from the left panel were analyzed. Co-localization was defined as any overlap between the two signals. The percentages of γ-H2AX foci with an overlapping 53BP1 signal are indicated. Right panel: 53BP1 foci from irradiated interphase cells in the left panel were analyzed for their co-localization with γH2AX as in the middle panel. One hundred and thirty-six distinct 53BP1 foci from 20 interphase cells were analyzed. During mitosis essentially no distinct 53BP1 foci were observed; thus mitotic cells were not included in this analysis. (C) U2OS cells were treated with DMSO or with the Plk1 inhibitor BI 2536 for 6 h. Anti-53BP1 and anti-γ-H2AX were used to stain DNA damage-induced foci. Average numbers of 53BP1 foci from 25 cells are indicated in the bar graph and representative cells with γ-H2AX staining are indicated. As a reference, U2OS cells were harvested 1 h after 5 Gy ionizing irradiation.
(1.19 MB EPS)

Figure S2 (A) Recombinant GST-Chk2 (1–219) was incubated with recombinant Plk1. GST-Chkl2 (1–219) was separated using SDS-page and subsequently purified and trypsin-digested. Phosphorylation of peptides was analyzed using LC-MS/MS. Phosphorylated serine and threonine residues and their relative position in a schematic Chk2 representation are indicated. (B) List of identified phosphorylated peptides. Observation frequency and observed phosphorylated residues are indicated. (C) Selection of phosphorylation sites. Identified phosphorylation sites that were observed at least twice and that showed an evolutionary conserved phosphorylation sites as well as a evolutionary conserved Plk1 phosphorylation consensus motif ([Asp/Glu][X][Ser/Thr]) are selected and depicted.
(0.69 MB TIF)

Table S1 For each indicated phospho-residue (column A), the conservation of the −5/+5 motif is indicated for 11 species (human-H.sap; rhesus monkey-M.mul; mouse-M.mus; rat-R.nor; cow-B.tau; dog-C.fam; platypus-O.ana; chicken-G.gal; African clawed frog-X.tro; zebrafish-D.rer; pufferfish-D.nig). Conservation is calculated and is indicated on a 0–1 scale. Full conservation of the −5/+5 motif results in a score of 1; absence of conservation or absence of the conservation of the phospho-residue results in a score of 0. “NA” indicates that sequence information for this species is unavailable. “Incomplete” indicates that gaps exist in the sequence data and that information for a specific residue could not be retrieved. Motif conservation (column “M”) indicates the mean conservation of the −5/+5 motif over all 11 species. Phosphosite conservation (column “N”) indicates the conservation rate of the actual phospho-residue.
(0.07 MB XLS)

## Abbreviations

53BP1: p53-binding protein-1
Asp: aspartic acid
ATM: Ataxia Telangiectasia-mutated
ATR: ATM and Rad3-related
BRCA1: breast cancer-1, early onset
Cdk1: cyclin-dependent kinase-1
Chk2: checkpoint kinase-2
DDR: DNA damage response
DSB: double strand break
FACS: fluorescence-activated cell sorting
FHA: forkhead-associated domain
GFP: green fluorescent protein
Glu: glutamic acid
GST: glutathione-S-transferase
IP: immunoprecipitation
IR: ionizing radiation
Nbs1: Nijmegen Breakage Syndrome-1
NHEJ: non-homologous end joining
PBD: Polo-Box Domain
PBS: phosphate-buffered saline
Plk1: polo-like kinase-1
SEM: standard error of the mean
Ser: serine
Thr: threonine
