# Supplementary figures and images for: A Mitotic Phosphorylation Feedback Network Connects Cdk1, Plk1, 53BP1, and Chk2 to Inactivate the G2/M DNA Damage Checkpoint
Source: PLoS Biol. 2010 Jan 26;8(1):e1000287. doi: 10.1371/journal.pbio.1000287 (PMC2811157; doi:10.1371/journal.pbio.1000287)

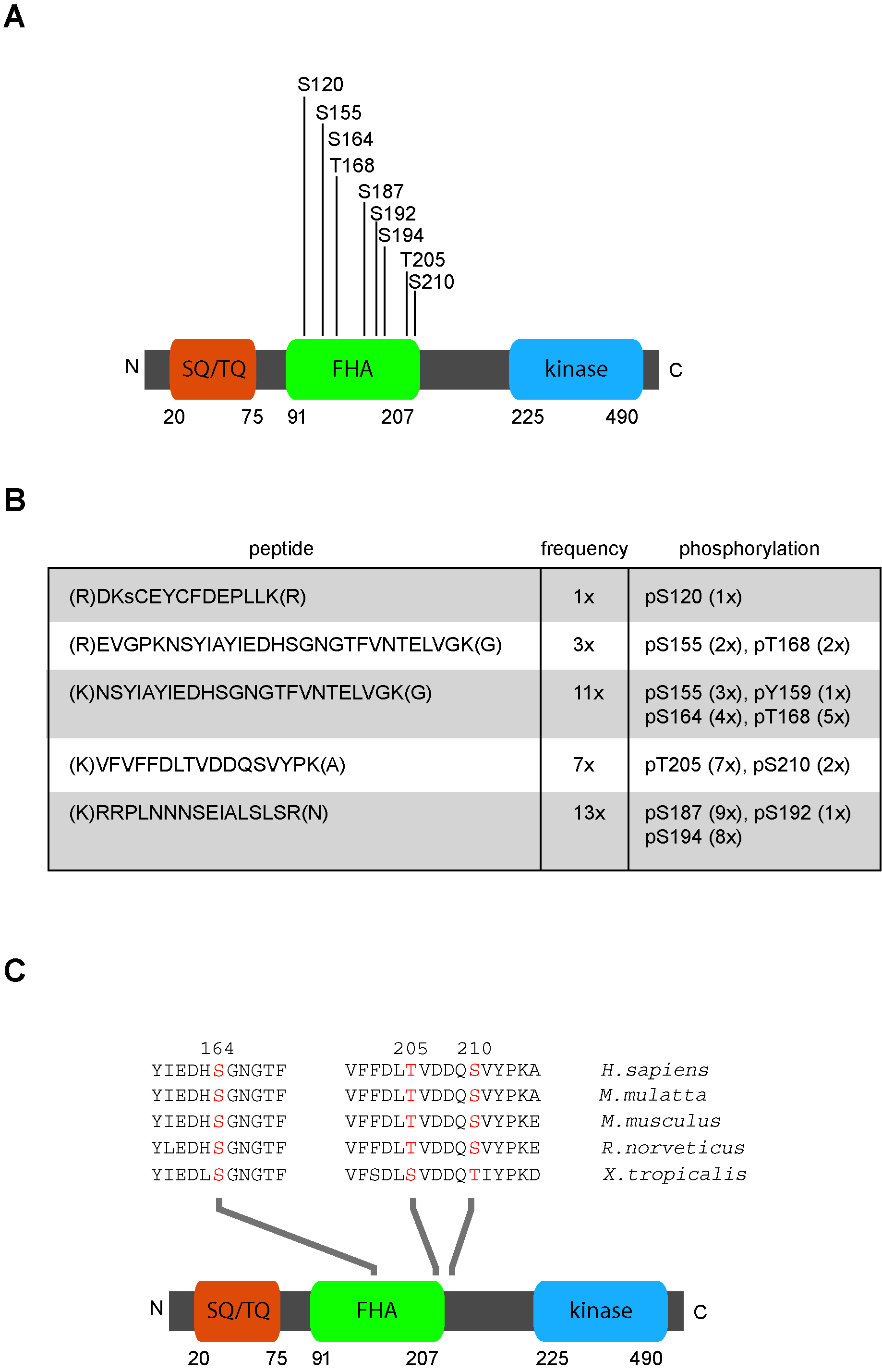

Supplement: Figure S2 — (A) Recombinant GST-Chk2 (1–219) was incubated with recombinant Plk1. GST-Chkl2 (1–219) was separated using SDS-page and subsequently purified and trypsin-digested. Phosphorylation of peptides was analyzed using LC-MS/MS. Phosphorylated serine and threonine residues and their relative position in a schematic Chk2 representation are indicated. (B) List of identified phosphorylated peptides. Observation frequency and observed phosphorylated residues are indicated. (C) Selection of phosphorylation sites. Identified phosphorylation sites that were observed at least twice and that showed an evolutionary conserved phosphorylation sites as well as a evolutionary conserved Plk1 phosphorylation consensus motif ([Asp/Glu][X][Ser/Thr]) are selected and depicted. (0.69 MB TIF) [file pbio.1000287.s002.tif]
